# Donor lamella thickness after ultrathin Descemet stripping automated endothelial keratoplasty and its relation to postoperative visual acuity and pre-operative lamella measures

**DOI:** 10.1186/s12886-023-03019-8

**Published:** 2023-06-12

**Authors:** Jeroen van Rooij, Angela Engel, Petra Steijger-Vermaat, Annemieke Molenaar, Juan Pedro Vigueras-Guillén, René J. Wubbels

**Affiliations:** 1 Rotterdam Eye Hospital, Schiedamse Vest 180, Rotterdam, 3011 BH The Netherlands; 2 Rotterdam Ophthalmic Institute, Schiedamse Vest 160, Rotterdam, 3011 BH The Netherlands; 3 Cornea Department, ETB-BISLIFE, Markt 58, Beverwijk, 1941 BM The Netherlands

**Keywords:** Corneal endothelium, Graft thickness profile, Edema

## Abstract

**Background:**

To accurately measure ultrathin Descemet stripping automated endothelial keratoplasty (DSAEK) donor lamella thickness during the first postoperative year and to correlate this with pre-operative and other postoperative measurements.

**Methods:**

Donor lamella thickness in 41 eyes undergoing DSAEK for Fuchs endothelial dystrophy (FED) was measured using the Tomey Casia OCT directly after graft preparation and at 1 week and 1, 3, 6 and 12 months postoperatively. Visual acuity and endothelial cell density were measured as the secondary parameters.

**Results:**

Individual graft thickness profiles were shown to be fairly regular within the optically relevant area. There was a strong and highly significant correlation between the pre- and postoperative lamellar thicknesses at all time points (p < 0.0001). Compared with the measurements directly after preparation at the cornea bank, the lamella thickness decreased by 12% after 12 months. Between 1 and 12 months postoperatively, the lamella thickness (mean ± SD) changed from 112 ± 27 μm to 101 ± 21 μm. Best spectacle-corrected visual acuity (BSCVA) changed from 0.46 ± 0.30 logMAR pre-operatively through 0.36 ± 0.33 at 1 month to 0.13 ± 0.16 at 1 year postoperatively. The endothelial cell counts were comparable to those reported in previous studies.

**Conclusions:**

Thickness profiles of individual grafts were fairly regular within the optically relevant area. A strong relationship between pre- and postoperative graft thicknesses was detected, and ultrathin DSAEK grafts prepared using methods similar to that applied in this study are expected to show a deswelling of around 12% during the first postoperative year. No correlation was detected between graft thickness and BSCVA.

## Background

Posterior lamellar keratoplasty (PLK) is currently the most frequently performed surgical technique for treating corneal endothelial cell dysfunction, predominantly caused by Fuchs endothelial dystrophy (FED) and pseudophakic bullous keratopathy. Compared to penetrating keratoplasty, PLK leads to better refractive results, with a faster recovery and improved safety [1, 2]. Currently, Descemet stripping automated endothelial keratoplasty (DSAEK) is the most frequently performed mode of PLK in both Europe [3] and the USA [4]. Although most randomized studies report a superior visual acuity (VA) after DMEK as compared to ultrathin DSAEK [4, 5], the robustness of this finding is not always evident [6], and DSAEK may be a better alternative in complex cases [7]. Moreover, a superior VA after either of these techniques is not always reflected into better patient reported outcomes [5].

While the donor lamellae for DSAEK were initially prepared by manual dissection, the introduction of an automated microkeratome for graft preparation [8] provided the possibility of preparing a smooth interface and to creating ultrathin grafts [9]. It was clearly demonstrated that, as compared to the ‘traditional’ microkeratome prepared lamellar grafts of around 200 μm, a better postoperative visual outcome can be accomplished after (depending on the criterion selected) 150 μm or 100 μm Ultrathin-DSAEK [10, 11].

For more than a decade, eye banks have stepped in to help surgeons with graft preparation of equally good quality [12], leading to efficient operating theatre use and avoiding the chance of donor tissue damage shortly before planned surgery. In tissue culture corneal donor preparation and storage many steps are undertaken, including swelling of the endothelial intracellular space for microscopic inspection and de-swelling of the corneal stroma in the transport medium [13]. Once transplanted into the recipient eye, the lamella was observed to shrink to a stable thickness in approximately 3 months [14]. The correlation between pre-and postoperative graft thickness has been studied before, but retrospectively, and using different techniques to measure pre- and postoperative graft thickness [15].

The aim of this prospective study was to investigate the relationship between pre- and postoperative posterior lamellar graft thickness in ultrathin DSAEK corneal grafts in subjects with FED and to evaluate postoperative deswelling. Pre-operatively and during 12 months after surgery the graft thickness was measured with the same anterior segment optical coherence tomography (AS-OCT) device. The secondary objectives of this study were to measure endothelial cells and study the relationship between graft thickness and visual outcome (best spectacle corrected visual acuity; BSCVA).

## Methods

### Patients

For this study, patients with FED and a visual acuity (VA) of < 0.6 (Snellen) were included. All subjects underwent ultrathin DSAEK at the Rotterdam Eye Hospital between May 2017 and July 2018. Exclusion criteria were previous keratoplasty in the included eye, severe progressive glaucoma (stable glaucoma on topical therapy was acceptable), history of retinal or glaucoma surgery, age-related macular disease, amblyopia, nystagmus, expected postoperative VA < 0.6 (Snellen), corneal neovascularization > 1 quadrant, or an indication for a tissue-typed graft. Ethical approval was waived by the Medical Ethical Committee of the Erasmus Medical Center, Rotterdam because the Dutch Medical Research Involving Humans Act (WMO) did not apply (study no.: MEC-2016-571, september 27th, 2016). This study adhered to the tenets of the Declaration of Helsinki. All the participants provided written informed consent.

### Donor preparation

All precut donor grafts were prepared in the same eye bank (ETB-BISLIFE, Cornea Department, Beverwijk, The Netherlands). The method of graft preparation and storage was described by Pels and Rijneveld [13]. After enucleation, the bulbi were decontaminated and stored in a culture medium containing antibiotics [10]. Macroscopic and slit lamp examinations were performed to evaluate preceding trauma, visible corneal disease, and previous surgeries. The corneoscleral button was excised and, after swelling of the endothelial cells in a hypotonic solution, examined under a light microscope. An evaluation of trypan blue-positive endothelial cells indicating cell death or damage and a cell count according to Gundersen’s method [16] was performed. The tissue was stored at 31 ˚C in the culture medium for a minimum of 6 days and a maximum of 4–5 weeks to allow for microbiological testing. Before preparation of the lamella, the endothelium was inspected once more with light microscopy, and the cornea was transferred to a culture medium with 6% dextran (Sigma Alfdrich, St. Louis, MO) for deswelling for 24–72 h. The precut graft was prepared by a single pass with a Gebauer SLc microkeratome (Gebauer Medizintechniek GmbH, Neuhausen, Germany) with a 300–600 μm range knife head and transferred back to the same medium with 6% dextran. Donor tissue was used within 3–7 days after transfer of the precut graft to its transport medium [13].

### Surgical procedure

The four cornea surgeons at the Rotterdam Eye Hospital performed all ultrathin DSAEK surgeries. After removal of the anterior cap and meticulous centering, the central donor graft was trephined with the endothelial side up using a vacuum donor cornea punch (Barron Precision Instruments, L.L.C., 8170 Embury Rd. Grand Blanc, MI, 48,439 USA) with a diameter of 7–8 mm (depending on donor and recipient cornea metrics). A superior 4.5 mm scleral tunnel was made in the recipient’s eye, and before opening the complete wound, a 7.5–8.5 mm descemetorhexis was performed using a Price hook (Moria, Antony, France) with the aid of air in the anterior chamber. A disposable type 2 Tan Endoglide™ (Angiotech Pharmaceuticals, Reading, USA) and a Tan forceps were used to insert the donor tissue. After 10 min of complete air filling of the anterior chamber, an air bubble of approximately the same diameter as that of the graft was left to keep the donor in place. Whenever possible, the marking made by the eye bank to indicate the cutting direction on the donor cornea was oriented horizontally in the recipient’s eye. Postoperatively, the patients were kept in the supine position for 3 h before they were discharged from the hospital.

After surgery, patients were prescribed dexamethasone (0.1%) eye drops (TEVA, Haarlem, The Netherlands) six times a day for the first month, four times a day for the following two months, and then tapered to one time daily at 12 months. Ofloxacin eye drops were administered 3 times daily for a maximum of 4 weeks.

### Measurements

Assessments were conducted pre-operatively and at 1, 4, 13, 26 and 52 weeks after surgery. Corneal images were acquired pre-operatively, with the posterior lamella still in situ, at the ETB-BISLIFE, Cornea Department, and postoperatively at our hospital using the same type of anterior segment (AS) OCT (CASIA SS-1000 Tomey, Nagoya, Japan). Images (16 mm x 16 mm) were acquired in both 2D and 3D modes (scan resolutions of 2048 and 512 lines, respectively). When the auto-alignment function indicated good alignment, three consecutive scans were performed. A scan for which the central reflective beam was visible on all images, warranting perpendicular recording, was used for the analysis.

One week postoperatively, only OCT images were recorded. From the first month postoperative onward, the following measurements were performed: AS-OCT, BSCVA (ETDRS chart, Precision Vision, Woodstock, USA; on a lightbox, Steinbeis Transferzentrum Biomedisinische Optik, Germany), and endothelial imaging using specular microscopy (Topcon SP-1P, Topcon Co., Japan). The best endothelial image was selected by observation for analysis with custom-made software [17]. Participants were requested to fill out the VFQ-39-NL visual function questionnaire (i.e. VFQ-25 including optional questions) at baseline and at 6 and 12 months.

### Analysis

Data were collected in Castor EDC (Ciwit BV, Amsterdam, The Netherlands) and analyzed using Microsoft Excel (2010) and IBM SPSS Statistics for Windows, version 23 (IBM Corp., Armonk, N.Y., USA).

As the pre- and postoperative (rotational) graft orientations may not be identical, 16 radial corneal images were used to construct a circular lamellar thickness profile of 32 values at 2 mm from the center: one profile from the pre-operative measurements and the other from the initial postoperative examination. Using cross-correlation, the angle of rotation for an optimal match of both profiles was determined for each graft.

For longitudinal analysis of the lamellar thickness, only two (matched) radial images were used, which were (arbitrarily) denoted as the ‘horizontal’ and ‘vertical’ profile respectively; each profile comprising 5 lamellar thickness values (i.e. corneal center, ± 1 mm and ± 2 mm distant from the center).

For analytical purposes, the pre-operatively obtained Snellen VA was converted to logMAR values. Regression analysis was performed to examine the relationship between the pre- and postoperative lamellar thickness and between lamellar thickness and visual acuity.

The VFQ-39-NL scores were recoded, and the composite outcome was calculated [18]. Because the number of matched questionnaires was limited, a t-test was performed for both the independent and matched data.

## Results

We included 47 eyes of 47 patients with Fuchs endothelial dysfunction in this study.

Due to missing data, particularly on pre-operative lamellar thickness, 6 patients were excluded from the analysis. The baseline characteristics are presented in Table 1. Seven patients underwent a re-bubbling procedure within 8–14 days after surgery.

**Table 1:** Baseline characteristics of study participants

Eyes (subjects) included (n)	47
Excluded from the analysis (n)	6
Eyes included in the analysis (n)	41
Male/Female (n)	24/17
OD/OS (n)	23/18
Age (years; mean ± SD) at transplantation	73 ± 7
Pre-operative CLT (µm; mean ± SD)	115 ± 23
Pre-operative VA (logMAR; mean ± SD)	0.46 ± 0.3
Pre-operative donor ECD (mm^− 2^; mean ± SD)	2698 ± 178

CLT: central lamellar thickness, VA: visual acuity, ECD: endothelial cell density

**Fig. 1:**
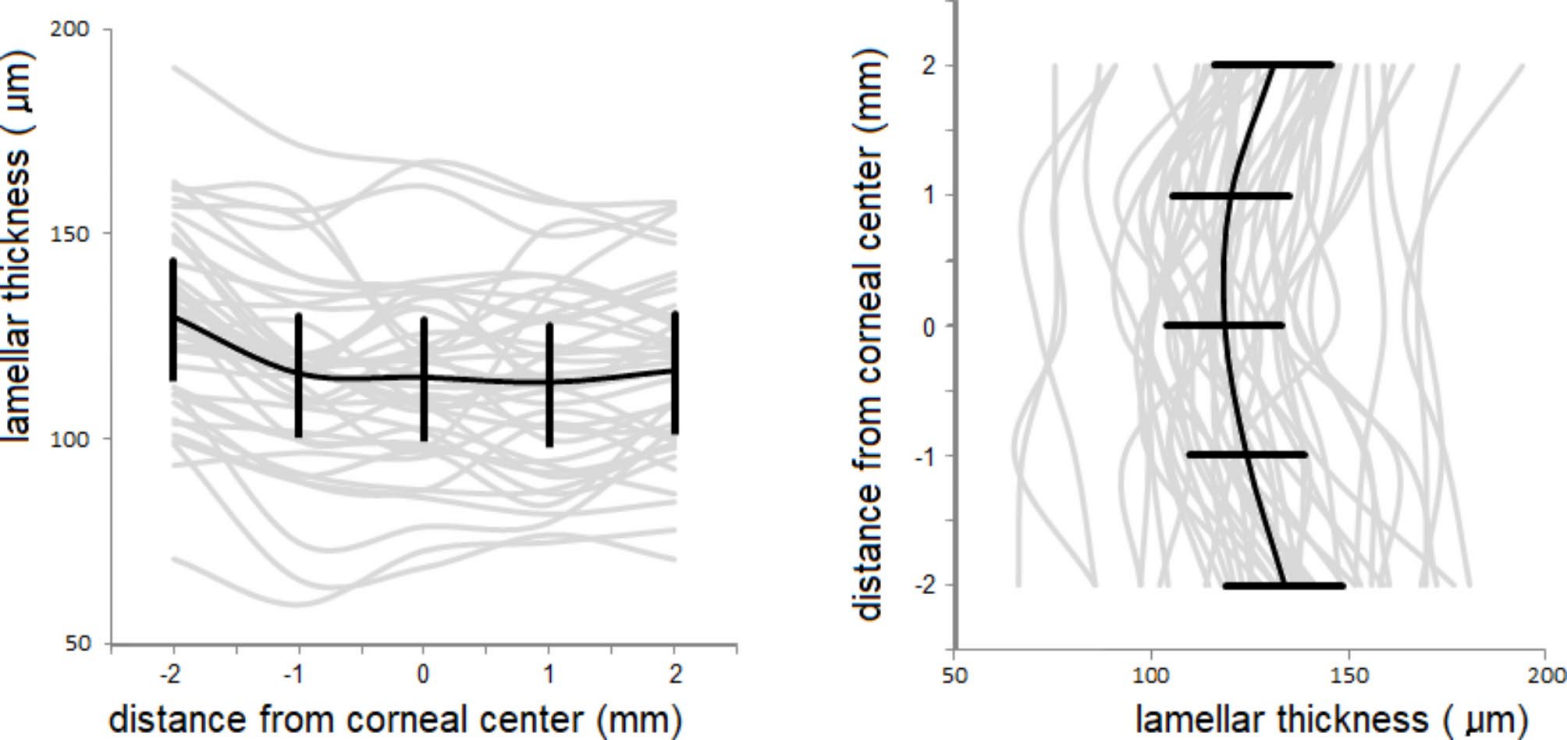
Pre-operative lamellar thickness profiles (grey lines) of 41 donor grafts in the horizontal (left) and vertical direction. Thickness was measured at the center and at 1 and 2 mm left and right of the center (horizontal) and at the center and at 1 and 2 mm above and below of the center (vertical). The solid black lines show the mean of the measurements at each position, error bars represent the 95% confidence intervals of the mean

**Fig. 2:**
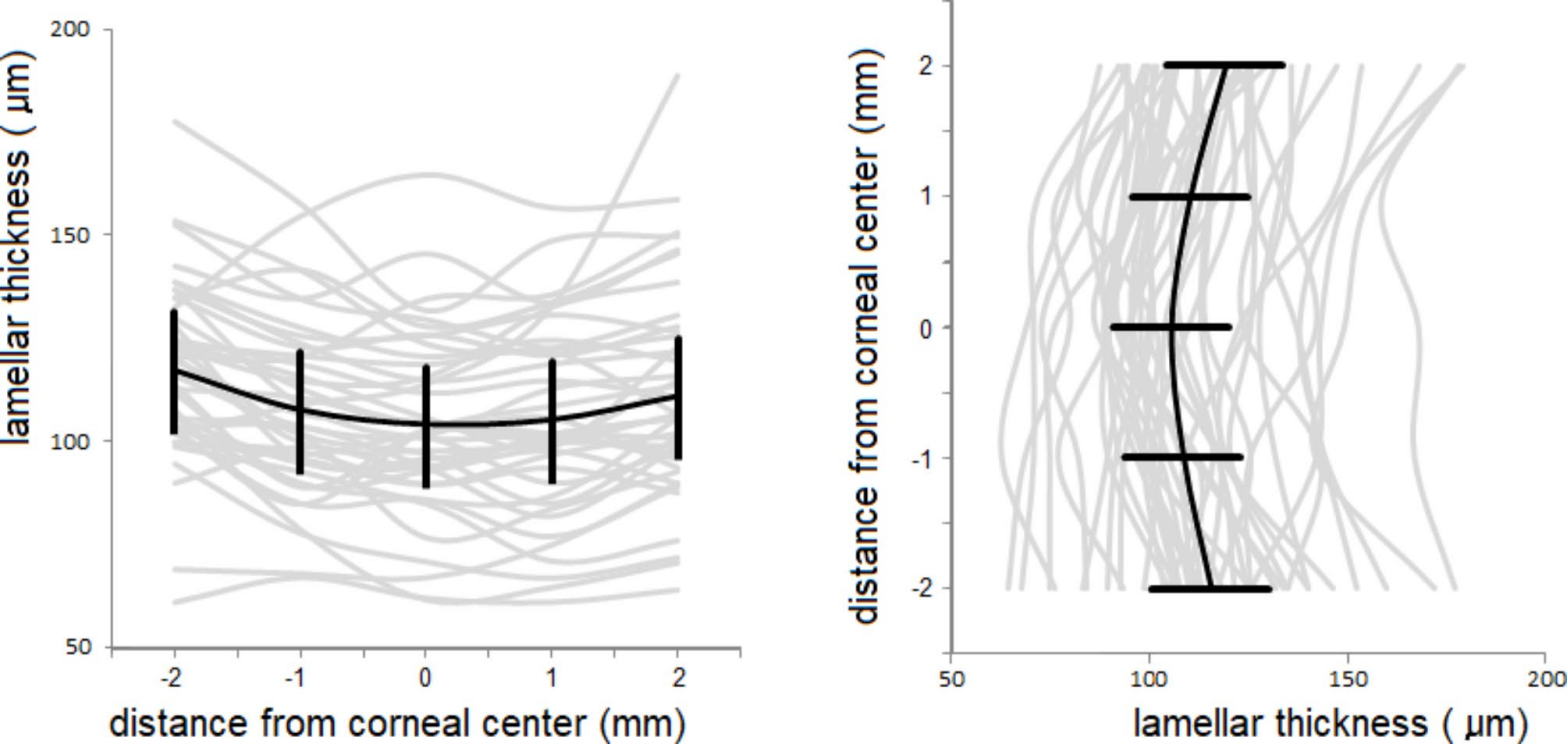
Lamellar thickness profiles of the same 41 donor grafts as in Figs. 1 at 26 weeks after surgery

The radial thickness profiles (i.e. at the five locations along the ‘horizontal’ and ‘vertical’ axes) of all 41 pre-operative posterior lamellae are shown in Fig. 1, together with their means. Although the variation between grafts is considerable, the lamellar thickness appears to be consistent within most individual grafts, indicating that the microkeratome cuts of the donor lamellae were regular in the optically relevant area. For comparison, the same profiles after six months are shown in Fig. 2. On average, the lamellae were thinner at the center than at the more peripheral locations.

**Table 2:** Postoperative outcomes of CLT (µm), VA (logMAR) and ECD (mm− 2)

Visit (weeks)	CLT	VA	ECD
1	136 ± 39 (34)		
4	112 ± 27 (41)	0.36 ± 0.33 (41)	1780 ± 458 (39)
13	106 ± 24 (41)	0.28 ± 0.41 (41)	1529 ± 538 (39)
26	104 ± 22 (41)	0.24 ± 0.35 (41)	1459 ± 510 (39)
52	101 ± 21 (40)	0.13 ± 0.16 (36)	1372 ± 491 (39)

Values are given as: mean ± SD (n)

CLT: central lamellar thickness, VA: visual acuity, ECD: endothelial cell density

**Fig. 3:**
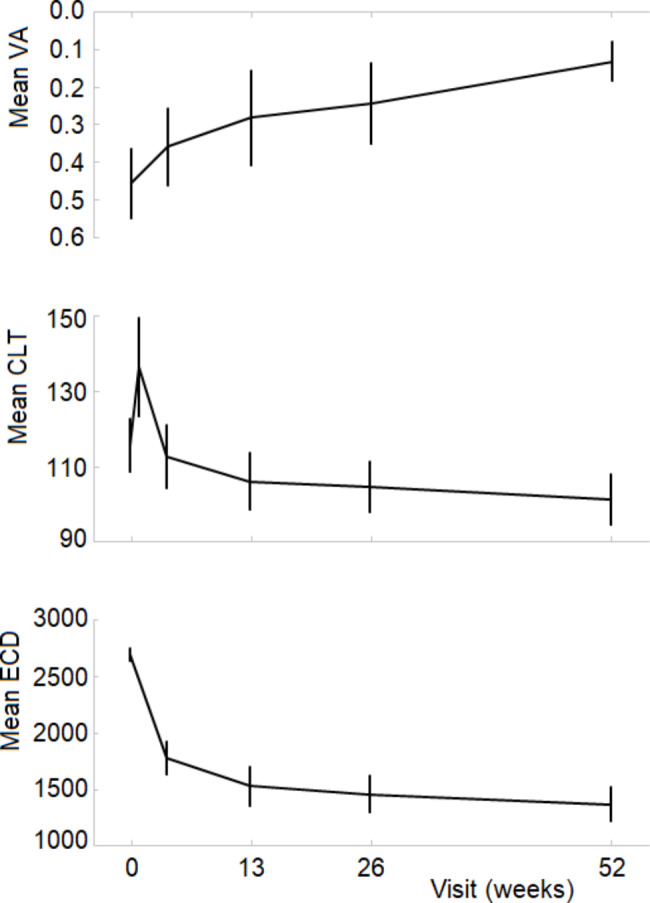
Pre-operative (week 0) and postoperative, visual acuity (VA, logMAR), central lamellar thickness (CLT, µm) and endothelial cell density (ECD, mm^− 2^) during follow-up. Error bars represent the 95% confidence intervals of the means

The longitudinal postoperative results with respect to central lamellar thickness (CLT), VA and ECD are summarized in Table 2; Fig. 3. After a sharp increase at 1 week postoperatively, the successively measured mean CLT’s were below the pre-operative mean, with the decline continuing up to the last measurement at 1 year. As expected, a strong and highly significant correlation was observed between pre-operative lamellar thickness and that measured at any postoperative visit. From 4 to 52 weeks postoperatively, Pearson’s correlation ranged from 0.73 to 0.76; P < 0.0001).

CLT was examined separately for grafts that had required rebubbling and for those that had not. The former decreased from a mean thickness of 135 ± 23 μm at baseline to 119 ± 25 μm at the end of follow-up, for the latter CLT was reduced from 111 ± 21 μm to 98 ± 19 μm; the difference at baseline being statistically significant (2-sided independent t-Test, P = 0.03) and at 52 weeks being not (P = 0.09).

**Fig. 4:**
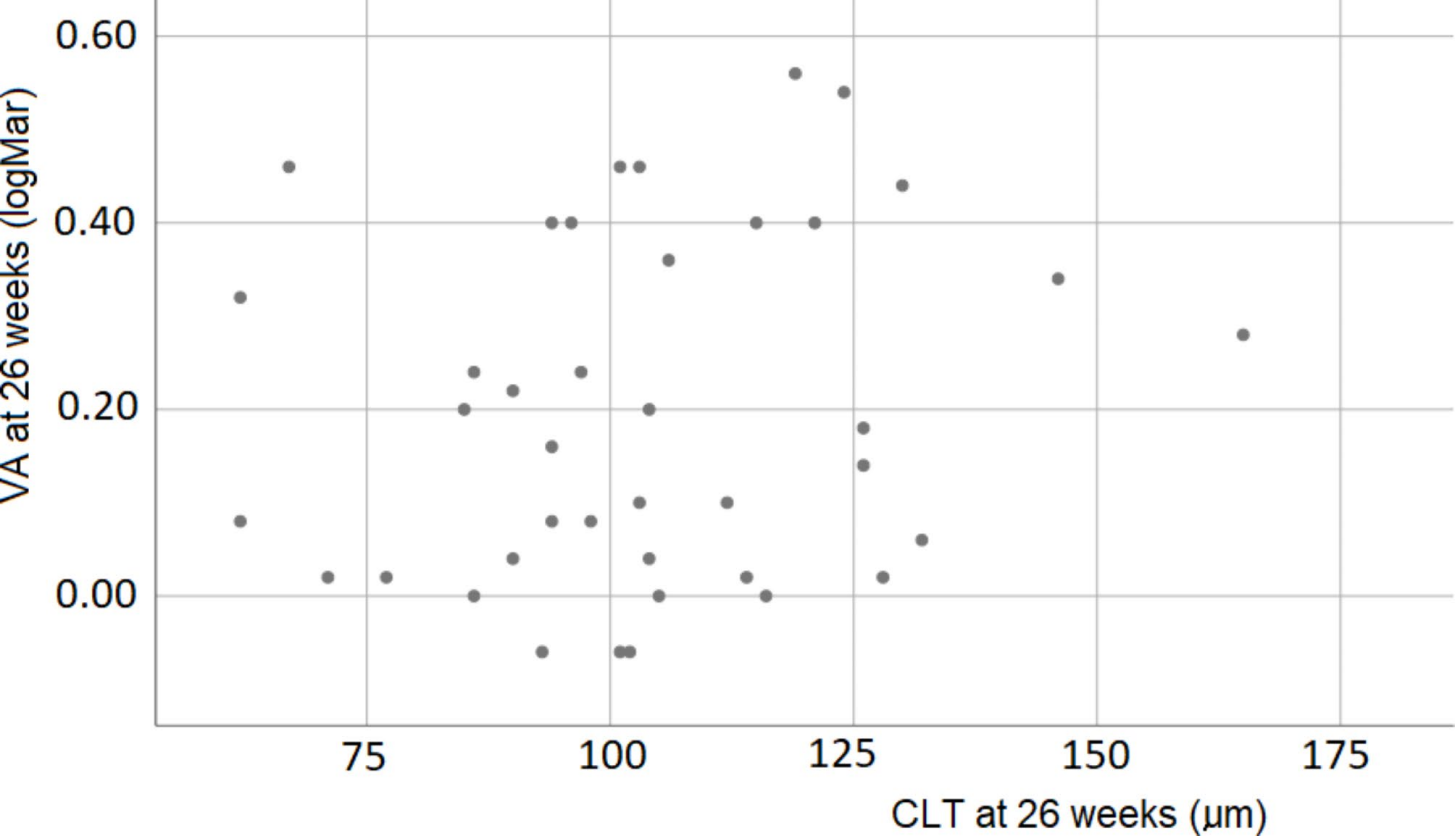
Visual acuity as a function of central lamellar thickness at 26 weeks (outlier, CLT: 135 μm, VA: 2.1 logMAR, not shown). No significant correlation was observed (n = 40, p = 0.28; outlier included, n = 41, p = 0.08)

During the entire follow-up period, ECD decreased, and VA improved. No significant correlation was observed between VA and lamellar thickness at any of the visits during follow-up visits (see the example in Fig. 4). After 26 weeks, VA of one patient was 2.1 logMAR (who also had worst pre-operative VA: 1.48 logMAR), VA of the other 40 subjects at that visit ranged from − 0.06 to 0.56 logMAR. The VFQ-39 scores improved significantly (Table 3). The correlation between lamellar thickness and visual function scores, however, was not significant.

**Table 3:** Visual function questionnaire scores: mean ± SD (n)

Visit (weeks)	VFQ-39 score	Independent t-Test	Matched t-Test
0	66.9 ± 12.8 (26)		
26	77.9 ± 13.3 (36)	p = 0.0018	0.0043 (23)
52	81.9 ± 10.2 (31)	p = 0.0000	0.0004 (18)

P-values relative to week 0; matched t-Test with number of pairs in analysis (n)

## Discussion

The aim of this study was to accurately describe the lamellar graft thickness during the first postoperative year after ultrathin (UT)-DSAEK and to analyze the correlations between pre-and postoperative graft thickness measurements.

Measurements of the posterior lamella with the aid of Casia OCT proved to be reproducible, and after correction for variations in rotational positioning of the grafts (see Methods) measurement positions at the donor tissue bank and postoperative measurements were matched. Compared to the pre-operative values, the thickness showed a steep increase at 1 week postoperatively. At one month, the lamellar thickness was slightly lower than the pre-operative values (Fig. 3, middle panel); from one month to 12 months, a continuing decline was observed. These results are in agreement with what has been reported by previous studies[19, 20]. The reduction of lamella thickness was paralleled by an improvement in visual acuity (Fig. 3, upper panel). From this, the possibility of an even further, though small, improvement after one year might be anticipated.

We demonstrated a strong correlation between the lamellar thicknesses as measured pre-operatively and the lamellar thickness at any postoperative time point. From this, it is concluded that after ultrathin DSAEK surgery, a 12% reduction in lamellar thickness at one year should be expected. This finding may aid in the clinical evaluation of DSAEK grafts. For example, in pachymetry measurements could be used to evaluate corneal edema after a rejection episode. A previous comparable but retrospective study [15], also reported a significant correlation between the pre- and postoperative graft thickness of DSAEK grafts. However, in another study, where significant deswelling of the donor cornea was achieved by anterior stromal dehydration with a flow of air before microkeratome-assisted DSAEK lamella preparation was performed, no significant postoperative thickness reduction was observed during the first postoperative year [21].

The small differences between the paracentral horizontal- and vertical thickness measurements indicate that the microkeratome-assisted DSAEK donor preparation in the tissue bank provides regular central cuts which is beneficial for the optical properties of the grafts.

While lamella thickness decreased by a modest 10% between 1 and 12 months postoperatively, mean VA continued to improve during this period of time from 0.36 to 0.13 logMAR. The relationship between lamella thickness and visual acuity has been the subject of a meta-analysis including 23 (cohort and case) studies [23], which resulted in a pooled correlation coefficient of 0.29 (95% CI, 0.16–0.41). At approximately the same time, results were published from a (multi-center randomized clinical) trial comparing DSAEK and ultrathin DSAEK [10], thereby covering a relatively wide range of graft thicknesses, with a highly statistically significant correlation (0.59) between lamella thickness and VA. It has been pointed out before, however, that such results do not automatically imply a clinically significant effect of graft thickness on visual acuity [22]; the improvement in VA was similar in both study arms (see Fig. 2 of Dickman et al. [10]). Neither in our retrospective study [22], nor in our present prospective study, we found any statistically significant correlation between lamella thickness and BSCVA. Although the correlation coefficients of all studies referred to above [10, 22, 23] and our present study were positive, the clinical significance of this statistically significant effect is rather poor. Moreover, we did not observe any significant correlation between lamella thickness and patient reported visual function.

## Conclusions

The thickness profiles of the individual grafts appeared to be fairly constant within the optically relevant area. In this prospective study, measuring pre-and postoperative ultrathin DSAEK graft thickness with the same type of OCT and using a strict measuring protocol, we demonstrated a strong relationship between pre-and postoperative graft thicknesses. It can be expected that such grafts will show a deswelling of approximately 12% during the first postoperative year compared to the thickness measurements directly after preparation.

## Data Availability

The dataset generated and analyzed for the present study is available from the corresponding author on reasonable request.

## Abbreviations

AS-OCT: Anterior segment OCT
BSCVA: Best spectacle corrected visual acuity
CLT: Central lamellar thickness
DSAEK: Descemet stripping automated endothelial keratoplasty
ECD: Endothelial cell density
FED: Fuchs endothelial dystrophy
PLK: Posterior lamellar keratoplasty.
